# A Large-Scale Agricultural Land Classification Method Based on Synergistic Integration of Time Series Red-Edge Vegetation Index and Phenological Features

**DOI:** 10.3390/s25020503

**Published:** 2025-01-16

**Authors:** Huansan Zhao, Chunyan Chang, Zhuoran Wang, Gengxing Zhao

**Affiliations:** National Engineering Laboratory for Efficient Utilization of Soil and Fertilizer Resources, College of Resources and Environment, Shandong Agricultural University, Tai’an 271018, China; 2021010046@sdau.edu.cn (H.Z.); chyan0103@sdau.edu.cn (C.C.); wangzr@sdau.edu.cn (Z.W.)

**Keywords:** Shandong province, agricultural land classification, time series, red-edge vegetation index, phenological features

## Abstract

Agricultural land classification plays a pivotal role in food security and ecological sustainability, yet achieving accurate large-scale mapping remains challenging. This study presents methodological innovations through a multi-level feature enhancement framework that transcends traditional time series analysis. Using Shandong Province, northern China’s agricultural heartland, as a case study, we first established a foundation with time series red-edge vegetation indices (REVI) from Sentinel-2 imagery, uniquely combining the normalized difference red edge index (NDRE_705_) and plant senescence reflectance index (PSRI). Moving beyond conventional time series analysis, we innovatively amplified key temporal characteristics through newly designed spatial feature parameters (SFPs) and phenological feature parameters (PFPs). This strategic enhancement of critical temporal points significantly improved classification performance by capturing subtle spatial patterns and phenological transitions that are often overlooked in traditional approaches. The study yielded three significant findings: (1) The synergistic application of NDRE_705_ and PSRI significantly outperformed single-index approaches, demonstrating the effectiveness of our dual-index strategy; (2) The integration of SFPs and PFPs with time series REVI markedly enhanced feature discrimination at crucial growth stages, with PFPs showing superior capability in distinguishing agricultural land types through amplified phenological signatures; (3) Our optimal classification scheme (FC6), leveraging both enhanced spatial and phenological features, achieved remarkable accuracy (93.21%) with a Kappa coefficient of 0.9159, representing improvements of 4.83% and 0.0538, respectively, over the baseline approach. This comprehensive framework successfully mapped 120,996 km^2^ of agricultural land, differentiating winter wheat–summer maize rotation areas (39.44%), single-season crop fields (36.16%), orchards (14.49%), and facility vegetable fields (9.91%). Our approach advances the field by introducing a robust, scalable methodology that not only utilizes the full potential of time series data but also strategically enhances critical temporal features for improved classification accuracy, particularly valuable for regions with complex farming systems and diverse crop patterns.

## 1. Introduction

Global agricultural crop production reached 9.49 billion tonnes in 2021 (Food and Agriculture Organization [FAO]), exhibiting pronounced spatial heterogeneity in its distribution. The Asian region dominated agricultural production, contributing 4.75 billion tonnes (50.1% of global output) and accounting for 45%, 72%, and 50% of global grain, vegetable, and fruit production, respectively. The remaining production was distributed among the Americas (2.51 billion tonnes; 26.5%), Europe (1.12 billion tonnes; 11.8%), Africa (0.99 billion tonnes; 10.4%), and Oceania (0.12 billion tonnes; 1.2%). At the national scale, agricultural production demonstrates significant concentration, with four countries—China, India, the United States, and Brazil—collectively contributing 54% of global cereal output. Among these nations, China maintains the leading position with an annual production of 720 million tonnes (23%), followed by India (12%) and the United States (11%). Within China’s agricultural landscape, Shandong Province serves as a cornerstone of the nation’s agricultural production system [1]. In 2023, the province’s grain production attained 55.8 million tonnes (8.2% of national output), comprising 24.8 million tonnes of wheat and 28.6 million tonnes of corn. The province’s vegetable production system encompasses 3.3 million hectares of cultivation area, generating an annual yield of 120 million tonnes (13% of national production), thereby making substantial contributions to China’s vegetable supply security. Furthermore, the province yields 22 million tonnes of fruits annually (12% of the national total). However, despite its agricultural prominence, Shandong Province faces substantial sustainability challenges, with access to merely 6% of China’s arable land and 1% of its freshwater resources, thereby imposing critical constraints on long-term agricultural sustainability.

Global food production currently faces two major challenges. First, there is mounting pressure from growing demand—approximately 800 million people still suffer from hunger and malnutrition [2], and according to FAO projections, current growth rates in food production are insufficient to eliminate hunger by 2030 or even 2050 [3]. Second, there is persistent environmental degradation, as the intensive pursuit of high crop yields has placed severe strain on land, water resources, and soil nutrients. Research has demonstrated that environmental issues such as soil degradation, soil erosion, climate change, and biodiversity loss pose serious threats to agricultural sustainability [4,5,6]. The intensification of global agricultural and ecological droughts has resulted in inevitable declines in crop yields [7]. In recent years, most countries have continued to rely heavily on increased resource inputs to boost production, such as excessive groundwater extraction and fertilizer use. However, these extensive farming practices have only exacerbated environmental problems. Addressing these challenges requires a fundamental shift towards resource-efficient utilization and scientific field management practices. Consequently, the ability to rapidly and accurately obtain information about agricultural land use patterns and their spatial distribution, combined with rational adjustment of crop structures, has become crucial for ensuring food security, protecting the environment, and achieving sustainable agricultural development [8].

Presently, a high-resolution map depicting the spatial distribution of various types of agricultural land in Shandong Province is unavailable. Hence, the exploration of a rapid and precise method for acquiring data on agricultural land types and implementing the monitoring and mapping of regional agricultural land’s spatial distribution is of paramount importance for the effective management of agricultural land use and safeguarding agricultural production.

Remote sensing technology, characterized by its objectivity, accuracy, real-time capability, cost-effectiveness, and high efficiency, has evolved as a critical technical tool for extracting spatial distribution information of land use types [9,10,11,12]. Due to the capability of multi-temporal remote sensing data to capture phenological variations in crops across different growth stages and the significance of vegetation indices in characterizing crop growth, the remote sensing classification of crops and land types utilizing multi-temporal or time series vegetation indices has garnered increasing attention from researchers [13,14,15,16,17,18]. As an illustration, Ignazio G et al. developed the modified soil adjusted vegetation index (*MSAVI_2_*) using the time series Sentinel-2 data and introduced a deep neural network-based technique for accurately mapping crop distribution at distinct time intervals [19]; Stefano M observed that the normalized difference vegetation index (*NDVI*) data, spanning from early tillering to spike stage across eight stages of Sentinel-2 satellite cluster analysis, produced crop distribution maps indicative of the impact of weed distribution on crop yield and its components [20]; Lukas et al. employed a random forest classifier (*RF*) to map crop types and sequences in Germany by integrating multi-year time series data from Sentinel-1, Sentinel-2, and Landsat 8. The study revealed that the classification approach incorporating optical, synthetic aperture radar (*SAR*), and environmental data exhibited strong spatial consistency and precise plot delineation, surpassing single-sensor methods [21]; and Hao discovered that the period from April to August in the time series yielded the highest accuracy in crop identification [22]. It can be seen that the classification accuracy can be effectively improved by exploring the optimal vegetation index and screening efficient time series features.

In comparison to conventional vegetation indices like NDVI and enhanced vegetation index (*EVI*), the red-edge vegetation index (*REVI*) exhibits greater sensitivity to variations in crop growth and phenology. Consequently, numerous researchers have initiated the use of multi-temporal or time series REVI for crop or agricultural land classification, aiming to achieve enhanced classification accuracy [23,24]. Kang et al. developed NDVI and ten distinct REVI time series utilizing GF-6 WFV data from Hengshui City at eight time steps, illustrating that integrating the normalized red vegetation index (*NDRE*) with NDVI enhances the accuracy and real-time efficiency of crop classification [25]; Vasileios et al. utilized the diverse spatial and time series vegetation indices derived from Sentinel-2 imagery to differentiate various crop types employing support vector machine (*SVM*) and random forest algorithms. Their research highlighted the enhanced performance of Sentinel-2’s spatial, spectral, and temporal characteristics in crop identification [26]; Wu et al. employed multi-temporal Sentinel-2A data to generate NDVI and NDRE time series for the detailed classification of crops in Jingtai County, China, validating that NDRE complements NDVI and enhances classification accuracy [27]. The aforementioned studies have shown that enhanced classification outcomes can be achieved by employing either a single REVI time series or a combination of non-red-edge and red-edge index time series for crop classification.

Unlike a single crop classification, crop types and their phenological characteristics are quite different from one agricultural land type to another; thus the complete fertility cycle of crops in each agricultural land has to be taken into account in the classification of farmland [28]. Some scholars have carried out relevant studies in this area, such as Agarwal et al., who used SPOT data to extract time series vegetation indices and their phenological characteristics to identify vegetation types in South Asia and achieved better classification results [29]. Kang et al. used 2-year time series MODIS EVI data to construct different vegetation phenology parameters to identify vegetation types in northeastern Inner Mongolia, and the results showed that the time series feature set combined with phenology characteristics could better distinguish different vegetation types [30]. Xiao et al. established the red-edge spectral index (RESI) based on the 2016–2018 Sentinel-2 time series data for crop identification in northern Laos’ Lang Namtha Province for remote sensing identification and mapping of rubber forests, proving that the RESI method combined with phenological features helps to improve the accuracy of classification [31]. The two REVIs, NDRE_705_ and PSRI, constructed by the Sentinel-2 red-edge band, can characterize the growth and decay status of vegetation in time series, which is important for the identification of crops and land types [32,33,34,35,36], and combining them with the phenological characteristics for the identification and extraction of agricultural land types deserves further research and exploration.

Most remote sensing studies on agricultural land distribution have focused on areas with simple topography at smaller spatial scales or specific crops with short growing cycles [21]. Research has shown that vegetation coverage exhibits high heterogeneity across different terrains and seasons, with diverse crop varieties leading to common “same spectrum–different object” and “different spectrum–same object” phenomena in remote sensing imagery, making crop classification particularly challenging in complex terrain and multi-seasonal contexts [37]. While Lam et al. and Cui et al. attempted to improve land cover classification accuracy in complex terrain through machine learning model optimization [38,39], using classification algorithms alone proved insufficient. Maung et al. achieved modest improvements using higher-resolution Sentinel-2 (10 m) imagery with a U-Net model for LULC classification in Myanmar’s Wunbaik mangrove region [40]. To further enhance classification accuracy in complex terrain, researchers began incorporating seasonal crop growth information into classification models [41,42]. Ma et al. proposed a U-Net (RIAU-Net) model based on the attention mechanism, which was applied to extract the rice planting information from Sentinel-1 images obtained in specific months [43]. Panchal et al. Panchal et al. analyzed different winter crop growth stages in India’s Vijapur Taluka using multi-temporal NDVI data to determine optimal periods for crop differentiation [44]. For regional-scale seasonal crop type identification using multispectral and SAR satellite imagery, Wittstruck et al. proposed a novel 3D deep learning U-Net model with squeeze-and-excitation fusion modules to progressively extract and combine image features, improving classification of spectrally similar and heterogeneous classes while mitigating cloud cover limitations [45]. Given Shandong Province’s diverse topography and distinct seasonal crop patterns, extracting accurate agricultural land distribution information through remote sensing remains a significant challenge.

Furthermore, due to the challenges in obtaining uniformly distributed classification samples on a large scale [46], conventional statistical methods for agricultural surveys remain prevalent at larger scales, posing difficulties in meeting the requirements for high-precision management and efficient utilization of agricultural land. Consequently, there is a pressing need to delve into rapid and accurate classification and mapping techniques for large-scale agricultural land types with complex topography and seasonal diversity [24,45,47,48].

This study addresses the challenge of large-scale agricultural land classification by selecting Shandong Province as the study area, constructing the time series REVI, integrating the spatial feature parameters (*SFPs*) and phenological feature parameters (*PFPs*), and employing machine learning algorithms to classify and map the major agricultural lands in the region. The objective is to enhance the accuracy of regional classification of agricultural land with complex topography and seasonal diversity through the comprehensive application of various remote sensing parameters, including time series REVI and phenology features. This study aims to develop a rapid and precise classification method for large-scale agricultural land types and achieve high-resolution spatial distribution mapping of these types, thereby providing a scientific foundation for the precise management and utilization of regional agricultural land.

## 2. Data and Methods

### 2.1. Overview of the Study Area

**Figure 1:**
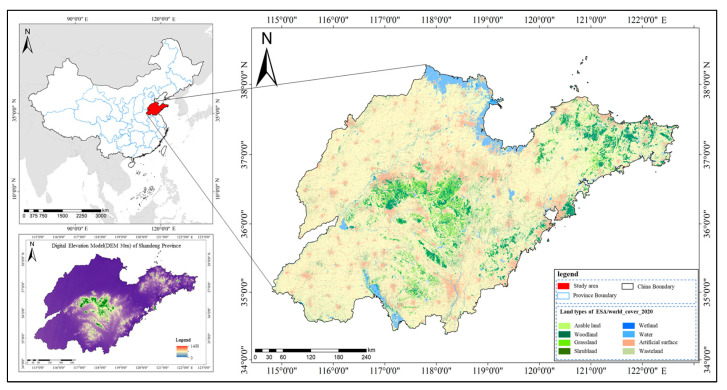
Overview of the study area. (The base map data is ESA/world_cover_2020, and the lower left corner displays the DEM of Shandong Province).

Shandong Province is situated between 114°36′ and 112°43′ E and 34°25′ and 38°23′ N, encompassing a total land area of 157,900 square kilometers. The province benefits from abundant light resources, with an annual average of 2290 to 2890 h of sunshine, and favorable heat conditions that support the cultivation of two crop cycles per year. As a major agricultural province, Shandong’s primary vegetation cover consists of crops, including winter wheat, summer maize, spring wheat, spring maize, cotton, peanuts, sweet potatoes, soybeans, fruit trees, and vegetables [49]. According to the Department of Agriculture and Rural Affairs of Shandong Province, the province’s grain output constituted 8.07% of the national total in 2019, while it ranked first in the country for both fruit and vegetable production, contributing 10.37% and 11.35% of the national totals, respectively. Among the major crops, the sown area of wheat was 4.00 million hectares, yielding a total output of 25.53 million tonnes. The sown area of corn covered 3.85 million hectares, producing 25.37 million tonnes, while vegetables were cultivated over 1.46 million hectares, resulting in 81.81 million tonnes. Additionally, the planted area of garden fruits was 585,340 hectares, with a total output of 17.40 million tonnes (Figure 1). Traditional agricultural survey and statistical methods are time-consuming and labor-intensive, making it challenging to meet the needs for efficient utilization and management of agricultural land. Therefore, there is an urgent need to develop and implement rapid and accurate agricultural land classification and mapping techniques.

### 2.2. Data Sources

#### 2.2.1. Sentinel-2 Data

Sentinel-2 is a high-resolution multispectral imaging satellite equipped with a Multispectral Imager (*MSI*) that covers a swath width of 290 km and offers a spatial resolution of 10 m. The Sentinel-2 constellation consists of two satellites, 2A and 2B, with an individual revisit period of 10 days. When combined, the two satellites provide a more frequent revisit period of 5 days. The imagery utilized in this study is sourced from the Level-2A (L2A) product dataset provided by the European Space Agency (*ESA*). The L2A data encompass red-edge spectral bands, which are highly effective for monitoring vegetation health indicators (Table 1).

This study conducted systematic processing of Sentinel-2 satellite data based on the Google Earth Engine (GEE) platform. Sentinel-2 MSI Level-2A (MSIL2A) data were selected as the primary data source, which underwent L1C to L2A conversion through the Sen2Cor processor, including atmospheric correction based on aerosol optical depth and water vapor, as well as terrain correction using SRTM 30m resolution DEM data. Image registration employed a multi-level strategy, combining Sentinel-2’s original geometric accuracy with the AROSICS sub-pixel registration algorithm, and accuracy validation was performed using evenly distributed ground control points to ensure registration errors were maintained within one pixel. The choice of L2A over L1C data was made because it has already undergone systematic atmospheric correction and directly provides bottom-of-atmosphere reflectance products, making it more suitable for time series analysis.

The study period chosen for this research spanned from September 2018 to October 2019, considering the quality of the Sentinel-2 imagery data source, the crop phenology periods of the agricultural land, and the availability of corresponding land use data during the same timeframe as the remotely sensed data. The L2A image dataset captured between September 2018 and October 2019 was obtained through online code editing on the Google Earth Engine (*GEE*) Remote Sensing Computing Service platform. This enabled the comprehensive coverage of the entire phenological cycle of crops across the study region. To efficiently address the effects of cloud cover, pixel-level de-clouding was performed utilizing the QA60 band and the scene classification (*SCL*) map available from the L2A dataset. First, data screening was performed based on cloud coverage thresholds: images with cloud coverage > 50% were eliminated, and images with cloud coverage > 20% were flagged. This study established a dual-layer cloud detection system based on the QA60 band and scene classification layer (SCL): The first layer utilizes QA60 band characteristics for coarse detection, where cloud detection and cirrus detection bits are 1 << 10 and 1 << 11, respectively. The second layer performs fine classification based on the SCL layer, identifying cloud shadows (class 3), medium probability clouds (class 8), high probability clouds (class 9), and cirrus clouds (class 10). Additionally, a 500-m dynamic buffer zone was set to reduce cloud edge effects. To optimize cloud detection performance under complex terrain conditions, this study adopted the following strategies: (1) dynamically adjusting cloud detection thresholds based on terrain features; (2) applying the Minaert model for topographic radiation correction; and (3) establishing a terrain shadow compensation mechanism. Due to the extensive coverage of the study area, a single timestamp image was insufficient to capture the entirety of the region. The study utilized a monthly time scale and adopted the median synthesis method to reconstruct time series images. Interpolation was performed to reconstruct based on phenological features for data-vacant areas. As all crops in the classified agricultural lands were in a dormant or stagnant growth phase from December 2018 to February 2019, resulting in less fluctuation in the corresponding vegetation index values, a single composite image was generated for this period. Subsequently, a time series image dataset encompassing 12 temporal phases in the study area was established (2018.09, 2018.10, 2018.11, 2018.12–2019.02, 2019.03, 2019.04, 2019.05, 2019.06, 2019.07, 2019.08, 2019.09, 2019.10). To ensure spatial resolution consistency, the reconstructed images were resampled to 20 m. Then, the Shandong Province administrative boundary vector data were loaded for image clipping, and the final dataset was stored in 32-bit floating-point format.

Crop area identification and monitoring under complex terrain conditions face multiple technical challenges. In mountainous and hilly areas, three main challenges exist: (1) spectral information distortion caused by terrain shadows; (2) changes in bidirectional reflectance characteristics due to terrain undulation; and (3) spectral heterogeneity resulting from terrain gradients. Additionally, mountainous farmland exhibits significant fragmentation characteristics, increasing identification difficulty. Plain areas present the following technical challenges: (1) spectral confusion between crops and natural vegetation; (2) heterogeneity in farmland plot scales; and (3) blurred plot boundaries.

For the above challenges, this study proposes the following solutions: (1) Classification image processing and plot optimization: applying morphological opening and closing operations to eliminate discrete noise; setting minimum plot area threshold (0.25 hectares); performing refined boundary extraction based on morphological gradient operators. (2) Adaptive parameter optimization for different terrain types: random forest classifier parameter settings (mountainous areas: 200 decision trees, minimum leaf node count 5; hilly areas: 150 decision trees, minimum leaf node count 4; plain areas: 100 decision trees, minimum leaf node count (3); Morphological operator window size (mountainous areas: 7 × 7 pixels; hilly areas: 5 × 5 pixels; plain areas: 3 × 3 pixels). (4) Establishing a multi-level accuracy assessment system, validating results through multi-dimensional evaluation metrics including Kappa coefficient, producer’s accuracy, user’s accuracy, and confusion matrix.

#### 2.2.2. NDRE705 and PSRI Time Series

NDRE_705_ [32,50,51] time series were calculated using the red-edge bands RE1 (B5) and RE2 (B6) from 12 time-phase L2A-level datasets. The formula is as follows:

(1) $${\mathrm{NDRE}}_{705}=\frac{\left(B6-B5\right)}{\left(B6+B5\right)}$$

The NDRE_705_ is an enhanced iteration of NDVI, renowned for its heightened sensitivity to chlorophyll content, subtle variations in leaf canopy, forest canopy openings, and vegetation senescence. It finds extensive utility in precision agriculture, forest surveillance, and identification of vegetation stress [50].

The PSRI [50] time series were computed using 12 time-phase L2A-level datasets in the red-edge band RE2 (B6), the 10 m spatial resolution red light band (B4), and the blue light band (B2). The formula is as follows:

(2) $$\mathrm{PSRI}=\frac{\left(B4-B2\right)}{B6}\;$$

PSRI enhances the sensitivity to the carotenoid-to-chlorophyll ratio, with a rise in PSRI indicating increased canopy stress, the initiation of vegetative senescence, and the maturation of plant fruits [50].

The two REVIs, NDRE_705_ and PSRI, depict the dynamics of vegetation growth and decay over time. Numerous studies have demonstrated a strong correlation between the temporal patterns of NDRE_705_ and PSRI and crop biophysical attributes, including photosynthetically active radiation absorption, vegetation coverage, and leaf area index [32,33,34,35,36].

#### 2.2.3. Classification Sample Data

In this study, we collected 300 sample points for each of the following areas: winter wheat–summer maize rotation area, single-season crop field, orchard, and vegetable field. Additionally, we collected 100 auxiliary sample points for forest and grassland, construction land, water bodies, and bare land. For specific acquisition methods, please refer to Section 2.5.

### 2.3. Technical Route of the Research

**Figure 2:**
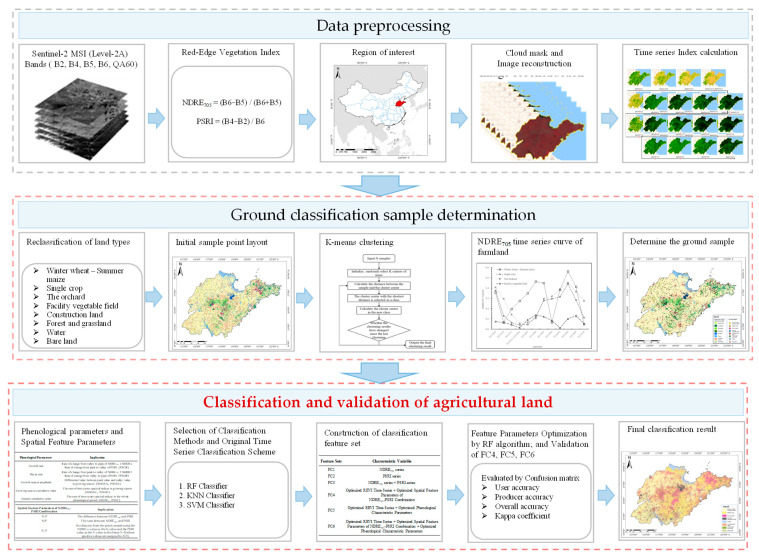
Technical route of the research.

Our study methodology comprised three interconnected steps (Figure 2) designed to achieve optimal agricultural land classification:

(1) Data Preprocessing:

We processed Sentinel-2 imagery on the Google Earth Engine (GEE) platform (the specific steps we expanded in Section 2.2.1) to construct two complementary vegetation indices: NDRE_705_ (normalized difference red edge index) and PSRI (plant senescence reflectance index). NDRE_705_ was selected for its proven sensitivity to chlorophyll content and canopy architecture, while PSRI effectively captured phenological transitions and vegetation stress responses. This dual-index approach enabled comprehensive characterization of vegetation dynamics throughout the growing season.

(2) Sample Collection and Optimization:

Our sampling methodology implemented a comprehensive multi-source validation framework, integrating K-means clustering analysis of NDRE_705_ temporal profiles with hierarchical reference data verification. The process began with temporal pattern identification from NDRE_705_ time series, which effectively captured distinct phenological signatures of different agricultural land types. These initial clusters underwent systematic cross-validation using multiple reference sources: high-resolution Google Earth imagery for visual interpretation of current land use patterns, 2019 land-use classification data for temporal consistency verification, 2020 global 10 m land cover data for spatial pattern confirmation, and local crop phenological calendars for growth cycle validation. Samples were retained only when showing consistency across at least three reference sources, while spatial stratification and minimum distance thresholds (>500 m) were applied to ensure representative coverage and minimize spatial autocorrelation. For specific acquisition methods, please refer to Section 2.5.

(3) Classification Implementation and Validation:

The classification methodology comprised three interconnected components. Initially, we optimized three machine learning algorithms (random forest, support vector machine, and K-nearest neighbor) using standardized accuracy metrics. We then developed two feature parameter sets: SFPs (spatial feature parameters) of NDRE_705_-PSRI Combination) for spatial characterization and PFPs (phenological feature parameters) for temporal characterization analysis. The final implementation combined the optimal algorithm with selected time series features and parameter sets to classify agricultural lands across Shandong Province. Validation using independent samples and confusion matrix analysis demonstrated robust classification performance while maintaining computational efficiency at regional scales.

### 2.4. Classification of Agricultural Land Types and Crop Phenological Information

**Figure 3:**
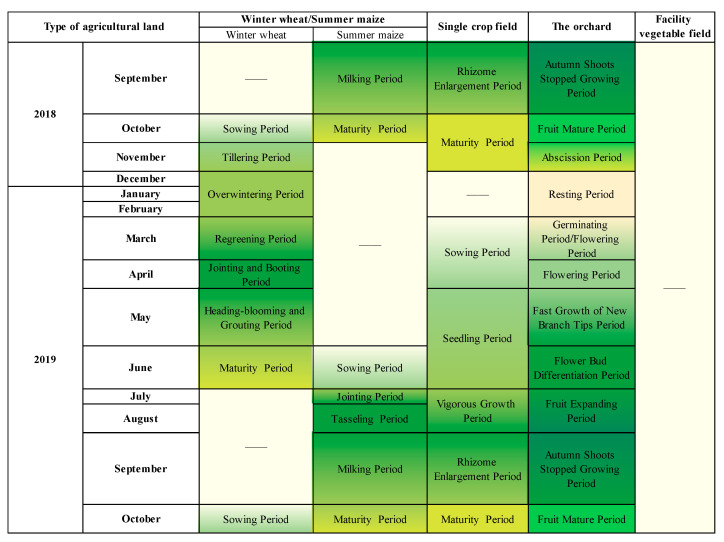
Phenological information of major agricultural lands in Shandong Province (crop calendar). (Summarization based on statistical information from the Department of Agriculture and Rural Affairs of Shandong Province [49]). The color changes represent the process of crop growth to decay.

The main types of agricultural land in the study area were classified based on the predominant crop types and the methods of land use, resulting in the identification of four primary agricultural land types: winter wheat–summer maize rotation area, single-season crop field, facility vegetable field, and orchard. These four agricultural land types are considered stable, long-term classifications in the study area, each characterized by specific cultivated crops, phenological traits, and production management strategies, encompassing grains, vegetables, fruits, and other crops, collectively occupying over 90% of the total agricultural land area in Shandong Province. These four types are typical and representative of agricultural land utilization in Shandong Province and the northern region of China. The phenological information of the four agricultural land types in Shandong Province from September 2018 to October 2019 is depicted in Figure 3.

### 2.5. Classification Samples Acquisition Methods

#### 2.5.1. Initial Sampling Point Acquisition

**Figure 4:**
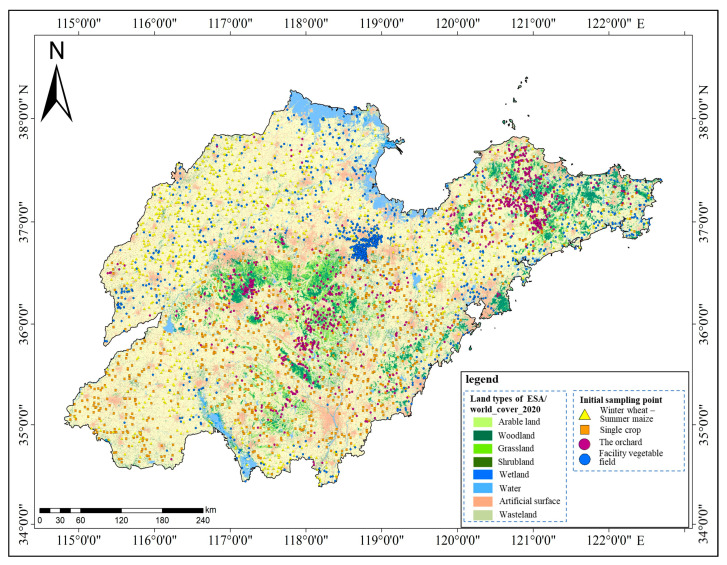
Schematic diagram of the initial sampling point deployment.

Initial sampling points were established utilizing the 2020 global 10 m ground cover data (obtained through the GEE platform) as the foundational map. Analysis of the base map data revealed that the three categories of agricultural land use in the study area (winter wheat–summer maize rotation area, single-season crop field, and orchard) were situated within the arable land category, whereas the facility vegetable field was situated within the wasteland category. Furthermore, overlay analysis of the 30 m digital elevation model (DEM) data (acquired through the GEE platform) and the global 10 m ground cover data in Shandong Province indicated that orchards were predominantly situated in areas with DEM > 100 m, single-season crop fields were located in areas with DEM > 50 m, and winter wheat–summer maize rotation areas and facility vegetable fields were mainly found in areas with DEM < 50 m. Based on the aforementioned analysis, coupled with data on the typical crop planting regions in Shandong Province, 1000 sampling points were strategically positioned. Specifically, 1000 sampling points were allocated from arable land with DEM < 50 m as the initial sampling points for the winter wheat–summer maize rotation area; another 1000 sampling points were designated from arable land with DEM > 50 m for the single-season crop field; an additional 1000 sampling points were deployed from arable land with DEM > 100 m for the orchard; and a further 1000 sampling points were placed from the wasteland with DEM < 50 m for the facility vegetable field, with a specific focus on Weifang and Liaocheng. To prevent overlap and ensure data integrity, the initial sampling points were set at a distance of 100 m apart (Figure 4).

#### 2.5.2. Determination of Classification Samples

The classification samples are primarily determined through the K-means clustering algorithm and the NDRE_705_ spectral curve characteristic of the target land type. Auxiliary datasets include Google Earth high-resolution imagery, 2019 land-use data, 2020 global 10-m ground cover data, and crop phenology information.

The NDRE_705_ curves of the initial sampling points were extracted, followed by K-means clustering analysis on the NDRE_705_ curves of the four types of agricultural land sampling points. The classification samples were determined from the clustering results in combination with auxiliary data, and additional sampling points were incorporated based on typical planting information. The final ground sample points must meet three criteria: (1) They must be located within the land classes corresponding to the 2020 global 10-m ground cover data and the 2019 land-use data, and visual interpretation must conform to the characteristics of the target land class; (2) the NDRE_705_ time series curves of the sample points must align with the trend of the mean curves determined by the K-means method; and (3) a buffer analysis (100 m) is conducted for the sample points that meet conditions 1 and 2, which is then superimposed on the 2020 global 10-m land cover data and the 2019 land-use data, ensuring that the area of the corresponding land classes within the buffer constitutes 80% or more of the total buffer area.

**Figure 5:**
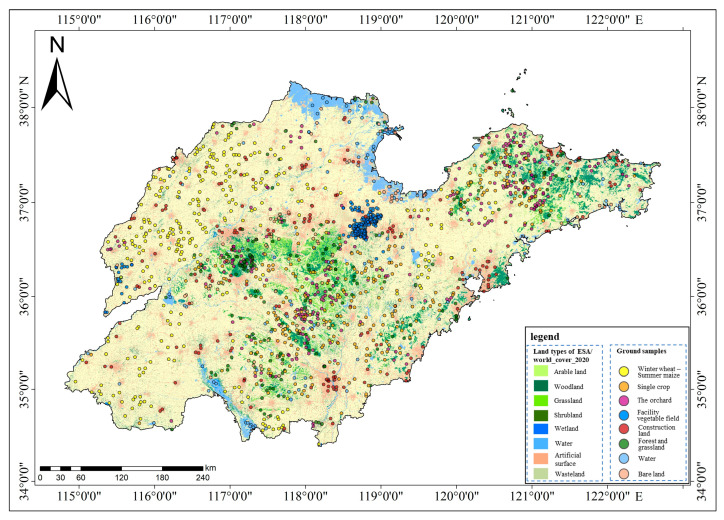
Classification sample distribution.

A total of 473 classification samples for the winter wheat–summer maize rotation area, 332 for single-season crop fields, 396 for orchards, and 308 for facility vegetable fields were initially obtained. Subsequently, 300 classification samples for each of the four types were selected for classification purposes. Additionally, 100 sample points for each of the four auxiliary categories (forest and grassland, construction land, water area, and bare land) were acquired using the same methodology. The distribution of classification sample points is shown in Figure 5. The training and validation sets were divided using a random sampling method in a 1:1 ratio.

### 2.6. Feature Parameters Construction and Optimization

In this study, the SFPs and PFPs were combined with the time series NDRE_705_ + PSRI to enhance the accuracy of agricultural land classification and identification.

#### 2.6.1. Spatial Feature Parameters of the NDRE_705_-PSRI Combination (SFPs)

The trends of the time series NDRE_705_ and PSRI curves vary among different crops, and the combination space of the two can significantly enhance the differentiation of information during crop growth at specific time points, thereby offering a foundation for the identification of agricultural land. In this study, three SFPs were selected, including difference space (N-P), ratio space (N/P), and dot pitch space (N_P).

The N-P and N/P spaces represent the difference and ratio, respectively, between NDRE_705_ and PSRI for the same agricultural land types at the corresponding time node. Additionally, N_P indicates the distance from the point formed by using the NDRE_705_ value as the X-value and the PSRI value as the Y-value to the linear Y = X (where X > Y is assigned positive values). The formula is outlined as follows:

(3) $$N-P={\mathrm{NDRE}}_{705}-\mathrm{PSRI}$$

(4) $$N/P\;={\mathrm{NDRE}}_{705}/\mathrm{PSRI}$$

(5) $$N\_P\;=\frac{\left|{\mathrm{NDRE}}_{705}-\mathrm{PSRI}\right|}{\sqrt{2}}$$

#### 2.6.2. Phenological Feature Parameters (PFPs)

This study employed the shape characteristics of the NDRE_705_ and PSRI time series curves to depict the phenological traits of various agricultural land types. The PFPs chosen for the investigation encompassed growth rate, decay rate, growing season amplitude, growing season cumulative value, and annual cumulative value, as detailed in Table 2.

#### 2.6.3. Evaluation and Optimization of Characteristic Parameters

In the context of remote sensing image classification, the redundancy of classification features frequently stems from the subjectivity inherent in feature selection. Feature optimization serves to mitigate the redundancy within classified data, diminish the impact of unreliable variables on classification outcomes, and enhance the accuracy of remote sensing classification [52].

In this study, we utilized the out-of-bag error (*OOB*) method, which is grounded in the RF algorithm, to evaluate the importance of the features requiring screening. The RF algorithm randomly selects n samples from the original sample set of N samples using sampling with replacement. Two-thirds of the original set’s samples are allocated to the decision tree training set, while the remaining one-third, not included in the selection, constitute the OOB set. The OOB set is leveraged to determine the importance of the features under scrutiny, facilitating the assessment of feature importance.

Employing the GEE platform to access the RF algorithm online, we conducted the evaluation and optimization of spatial feature parameters for the NDRE_705_-PSRI combination and PFPs. These processes laid the groundwork for enhancing the accuracy of agricultural land classification.

### 2.7. Classification Feature Set Construction

Six classification feature sets were assembled by integrating the time series NDRE_705_ and PSRI datasets with SFPs and PFPs, resulting in the creation of three original time series REVI feature sets (FC1, FC2, FC3) and three feature sets (FC4, FC5, FC6) combining the selected feature parameters with the original time series REVI (detailed in Table 3).

### 2.8. Classification and Validation

In this study, three classification methods, namely RF, SVM, and KNN, were employed for the classification of agricultural land types.

Three classifiers were implemented by online code editing on the GEE platform to achieve the recognition of agricultural land types based on the three classification methods, followed by the evaluation of recognition accuracy.

The accuracy of the classification system was assessed utilizing a confusion matrix that included indicators such as overall accuracy (*OA*), Kappa coefficient, producer accuracy (*PA*), and user accuracy (*UA*). By comparing the accuracies of various classification methods, the optimal system and model for agricultural land classification in the study area were identified.

### 2.9. Uncertainty Assessment

Our study implemented effective mitigation strategies for uncertainty through time series red-edge vegetation index features, robust phenological indicators, and terrain-adaptive parameter optimization. But field validation represents a critical component in ensuring the accuracy of crop phenological identification and agricultural land classification. Although this study established a systematic accuracy verification framework, comprehensive field validation was constrained by the extensive study area (approximately 158,100 km^2^), which would require substantial human and material resources for thorough ground-truthing. The limited field validation introduces certain uncertainties in classification results, primarily attributable to cloud coverage, complex terrain characteristics, and agricultural land fragmentation. Future research endeavors could systematically strengthen field validation through the integration of mobile data collection systems, high-resolution satellite remote sensing, and unmanned aerial vehicle (UAV) remote sensing technologies, thereby reducing classification uncertainties.

## 3. Results

### 3.1. NDRE_705_ and PSRI Original Time Series Curves for Different Agricultural Land Types

**Figure 6:**
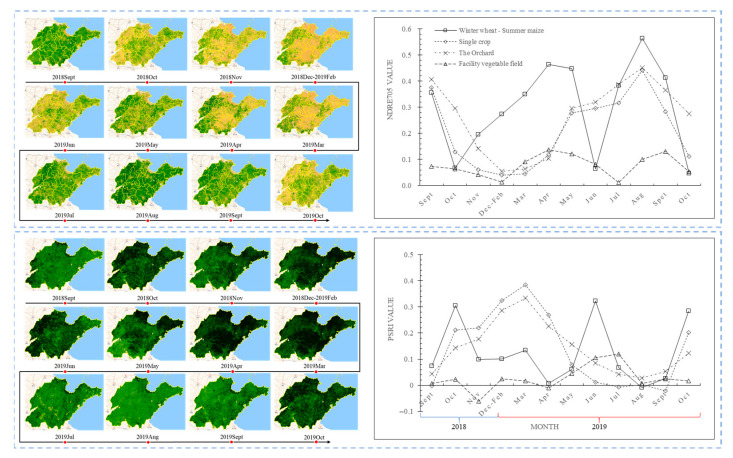
Visualization of NDRE_705_ and PSRI time series curves of different agricultural land types in Shandong Province.

The monthly reconstructed L2A image set was utilized to calculate and extract NDRE_705_ and PSRI values from the classification sample time series of four agricultural land types. The average values were then computed to derive the NDRE_705_ and PSRI mean curves for each agricultural land type over time. Subsequently, the monthly NDRE_705_ and PSRI mean values were visualized (Figure 6). It is observed that the mean value curves of NDRE_705_ and PSRI for each agricultural land type exhibit opposing trends. Both indices effectively capture the phenological variations associated with different agricultural land types across months and seasons.

By comparing the NDRE_705_ and PSRI curves for each type, it was found that both time series curves in the winter wheat–summer maize rotation area were bimodal, which could be easily differentiated from other agricultural lands. Single-season crop fields and orchards have similar curve trends, with both reaching their peaks in the same month. The NDRE_705_ curve reached its peak in August, while the PSRI curve reached its peak in March. However, the growth cycle of single-season crops is short, resulting in rapid changes in NDRE_705_ and PSRI values at specific time points. For instance, in September–October, when single-season crops reach maturity and are ready for harvesting, the NDRE_705_ value decreases rapidly while the PSRI value increases rapidly. During this time, the orchard begins to lose its leaves, resulting in a change in canopy color but without complete leaf shedding. The NDRE_705_ and PSRI values show minimal changes and remain within the range observed for green vegetation. Additionally, due to mulching, the time series NDRE_705_ and PSRI values for the facility vegetable field consistently fall within a lower range, with minimal fluctuations, making it easily distinguishable from other land types.

### 3.2. Selection of Classification Methods and Original Time Series Classification Scheme

**Figure 7:**
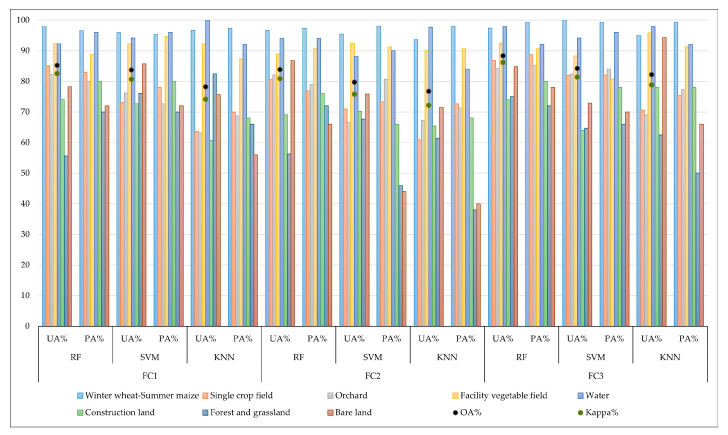
Accuracy of agricultural land classification for different original time series classification schemes.

Figure 7 presents the evaluation of agricultural land classification accuracies using three original time series classification schemes: NDRE_705_ (FC1), PSRI (FC2), and NDRE_705_ + PSRI (FC3), assessed by three classification methods: RF, SVM, and KNN. The NDRE_705_ + PSRI time series demonstrates the highest classification accuracy, followed by the NDRE_705_ time series, with the PSRI time series showing the lowest accuracy. Among the three classification methods, RF outperforms SVM and KNN. Specifically, the classification set utilizing the RF algorithm and the NDRE_705_ + PSRI original time series achieves the highest accuracy, with an overall accuracy (OA) of 88.38% and a Kappa coefficient of 0.8621. For various agricultural land types, the recognition accuracy was highest for the winter wheat–summer maize rotation area. Consequently, the RF classifier exhibited the best performance in land use classification with extensive feature information. Moreover, the combination of NDRE_705_ and PSRI time series effectively enhanced the recognition accuracy of agricultural land types.

### 3.3. Characterization Parameters Optimization

#### 3.3.1. Optimization for Spatial Feature Parameters of NDRE_705_-PSRI Combination (SFPs)

To verify the separability of N-P, N/P, and N_P for agricultural land types, the RF classifier was employed to classify agricultural land types using three spatial features. The overall accuracies (OA) were 81.50%, 83.63%, and 79.25% for N-P, N/P, and N_P, respectively, with corresponding Kappa coefficients of 0.7809, 0.8062, and 0.7537. The results indicate that although the time series N-P, N/P, and N_P can be utilized to classify agricultural land, their classification accuracies are all lower than that of the NDRE_705_ + PSRI time series. In light of these results, the study aims to prefer the eigenvalues of N-P, N/P, and N_P to assist the NDRE_705_ + PSRI time series in agricultural land classification and to explore the impact of the preferred SPFS on the classification accuracy of agricultural land types.

**Figure 8:**
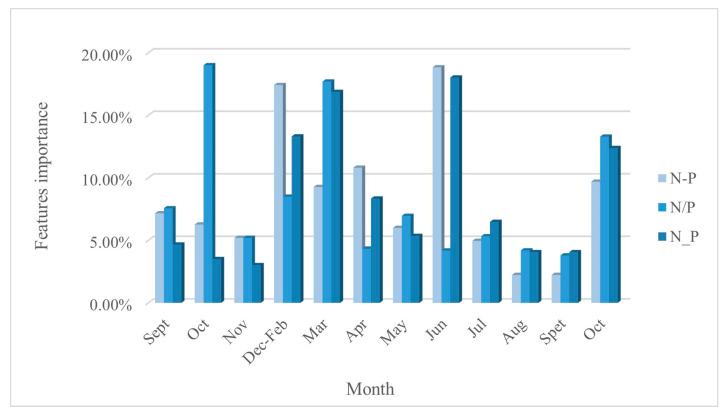
Feature importance of SFPs. (It shows the feature importance of the three SFPs in each time phase).

The feature importance of the three SPFS, N-P, N/P, and N_P, was evaluated using the GEE platform by invoking RF classifiers (Figure 8). The two features with the highest contribution rates for each spatial feature were selected as the preferred features. The preferred features for N-P were N-P (Dec-Feb) and N-P (Jun), contributing 17.42% and 18.83%, respectively. For N/P, the preferred features were N/P (Oct 2018) and N/P (Mar), contributing 19.01% and 17.70%. Lastly, for N_P, the preferred features were N_P (Mar) and N_P (Jun), contributing 16.87% and 18.02%, respectively.

#### 3.3.2. Optimization of Phenological Feature Parameters (PFPs)

**Figure 9:**
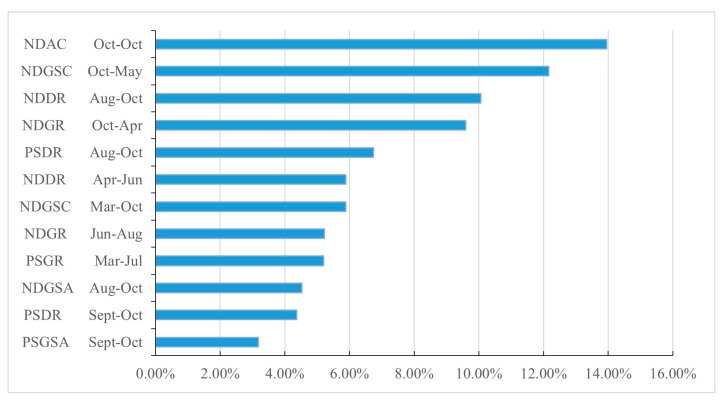
The 12 PFPs with the highest contribution. (Arranged in descending order of importance).

Five types of PFPs were calculated for each agricultural land type: growth rate, decay rate, growing season amplitude, growing season cumulative value, and annual cumulative value. In total, 32 PFPs were constructed (Table 4). Subsequently, the RF classifier was employed to classify the agricultural land using these 32 PFPs, and the classification accuracy was evaluated. The results indicated that OA of agricultural land classification based on PFPs was 85.63%, with a Kappa coefficient of 0.8297. This suggests that although it is feasible to utilize a collection of PFPs for agricultural land classification, its accuracy is lower than that of the NDRE_705_ + PSRI time series set. In this study, the 12 PFPs with the highest contribution rates (cumulative contribution rate of 86.82%) were selected to combine with the NDRE_705_ + PSRI time series for the identification of agricultural land types (Figure 9).

### 3.4. Time Series Classification Scheme Assisted by Preferred Features

The six spatial feature parameters of the NDRE_705_-PSRI combination and twelve PFPs were integrated with the NDRE_705_ + PSRI time series to create new classification feature sets, designated as FC4, FC5, and FC6. These sets were then used to classify agricultural land using the RF classifier. The classification accuracies are presented in Table 5.

As shown in Table 5, the classification accuracies of agricultural land in Shandong Province based on feature sets FC4, FC5, and FC6 surpassed that of the original NDRE_705_ + PSRI time series classification set FC3. Among them, the classification set FC4 with the addition of SFPs improved OA by 1.12% and the Kappa coefficient by 0.0136 compared to FC3. The classification set FC5 with the addition of PFPs improved OA by 2.00% and the Kappa coefficient by 0.0237 compared to FC3, indicating that PFPs are more effective than SFPs in enhancing the accuracy of agricultural land classification. The classification set incorporating both SFPs and PFPs achieved the highest classification accuracy, with an OA of 93.21% and a Kappa coefficient of 0.9159, representing increases of 4.83% and 0.0538, respectively, over the original time series classification set FC3. This demonstrates that the combination of preferred SFPs and PFPs with the NDRE_705_ + PSRI time series can effectively enhance the accuracy of agricultural land classification in Shandong Province.

**Figure 10:**
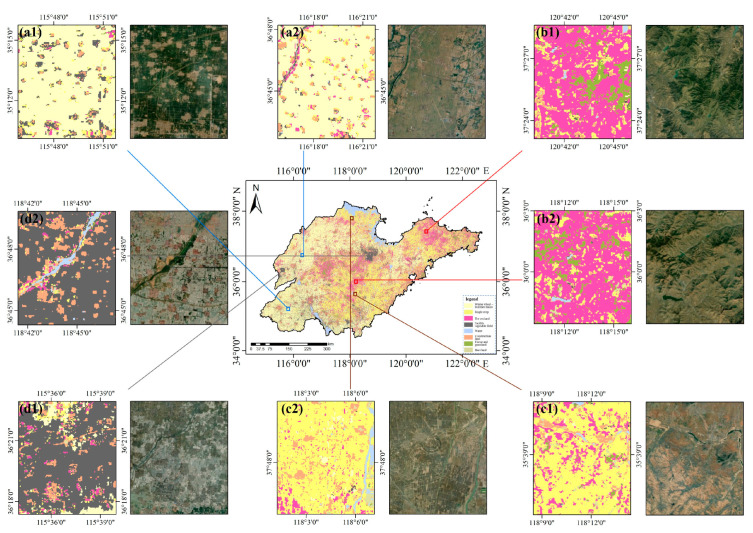
Details of the classification of different types of agricultural land. (**a1**,**a2**) Winter wheat–summer maize rotation area; (**b1**,**b2**) Orchard; (**c1**,**c2**) Single-season crop field; (**d1**,**d2**) Facility vegetable field. Note: Imagery is a high-resolution Google Earth image from October 2019.

Figure 10 illustrates the results and classification details of agricultural land classification based on feature set FC6 using the RF classifier. Two typical recognition areas were selected for each agricultural land type, combined with contemporaneous high-definition imagery from Google Earth (October 2019), to show the details. The recognition accuracy of the winter wheat–summer maize rotation area was the highest among the four agricultural land types, with a UA of 98.67%, followed by the facility vegetable field with a UA of 95.97%. These two types of agricultural land plots were more complete and aligned better with actual field boundaries. The UA for the single-season crop field and the orchard were 94.59% and 91.45%, respectively. As observed in the figure, the plots for these two types of agricultural land were relatively fragmented, with some overlap.

### 3.5. Area and Distribution of Agricultural Land Types in the Study Area

**Figure 11:**
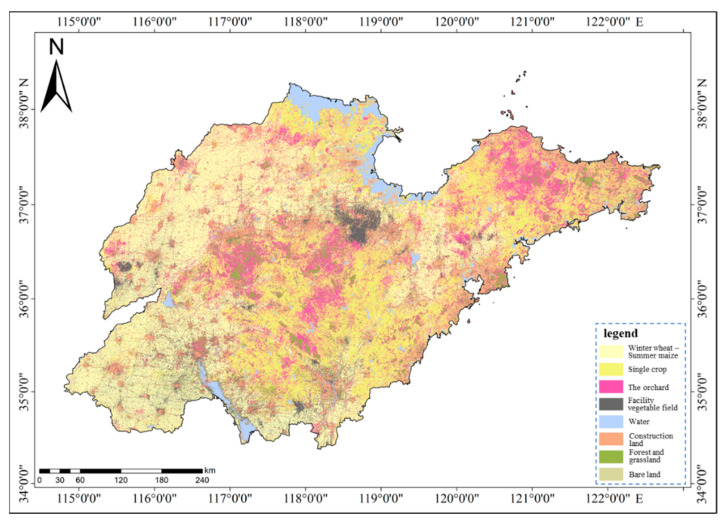
Distribution of agricultural land types in Shandong Province.

Figure 11 illustrates the distribution map of agricultural land in Shandong Province in 2019, ultimately derived from the feature set FC6 utilizing the RF classifier. Statistically, 120,996 km^2^ of agricultural land were identified. Among them, the winter wheat–summer maize rotation area covered 47,727 km^2^ (39.44%), the single-season crop field spanned 43,751 km^2^ (36.16%), the orchard encompassed 17,527 km^2^ (14.49%), and the facility vegetable field accounted for 11,991 km^2^ (9.91%). The winter wheat–summer maize rotation areas are predominantly situated in plain regions such as the Northwest Lu Plain Area, the South Central Lu Plain, and the North Lu Plain Area. Single-season crop fields are primarily found in areas with lower soil fertility levels, including the mountainous region in central Shandong, the Jiaodong hilly area, and the salinized area in northern Shandong. Orchards are mainly distributed in the mountainous regions of central Shandong and the hills of Jiaodong, with fewer yet more concentrated orchards in the plains, such as jujube orchards in Zhanhua and pear orchards in Guanxian. Facility vegetable fields are concentrated in Shouguang, Changle, Qingzhou, and other areas in Weifang City and are also more prevalent in Shen County of Liaocheng City and Cangshan County of Linyi City.

## 4. Discussion

(1) This study successfully classified the primary agricultural land types in Shandong Province by integrating time series NDRE_705_ and PSRI (Sentinel-2) data with phenological characteristics. The overall accuracy of the classification reached 93.21%. Moreover, the delineated boundaries of agricultural land closely matched the actual field boundaries, demonstrating a high level of agreement with the land cover classification results reported by Dong et al. [53] in Shandong Province. The effectiveness of integrating phenological characteristics with the REVI time for agricultural land classification was confirmed. It is important to acknowledge that the type and distribution of agricultural land are significantly influenced by factors such as temperature, moisture, and other climatic variables, leading to substantial variations in agricultural land types across different regions. While this approach offers a valuable reference for the broader-scale classification and mapping of agricultural land types, its suitability for classifying agricultural land in other provinces of China and at varying latitudes requires additional verification through practical implementation.

(2) Sufficient and precise classification samples are essential for ground cover classification [54], yet acquiring ground samples becomes challenging in large study areas. In this study, we introduced a rapid ground sample acquisition approach utilizing the K-means algorithm and the NDRE_705_ curve of the specific ground class. The study effectively minimizes human and material resources while ensuring the accuracy, sufficiency, and even distribution of ground samples. In contrast to the hexagonal automatic sampling strategy employed by Fu et al. [55], this study utilized the time series REVI characteristics to identify classification points, potentially enhancing the accuracy of acquired sampling points, albeit introducing subjectivity in their assessment. Further research is needed on automatically differentiating a small subset of sampling points with similar features.

(3) The selection and utilization of suitable feature variables are crucial for enhancing the precision of crop remote sensing classification [56,57], with time series vegetation index features showing a higher potential for enhancing land cover classification accuracy [58,59]. This study fully accounted for the variations in the time series of red-edge spectral features among diverse agricultural lands, employing a combination of two time series, NDRE_705_ and PSRI, exhibiting distinct trends for agricultural land recognition. Consequently, the accuracy of agricultural land classification surpassed that achieved with a single index, as the NDRE_705_ + PSRI time series can comprehensively capture crop growth status from various angles, containing a richer set of feature information. A comparative analysis of three classification methods demonstrated the superiority of the random forest (RF) algorithm in classifying surface cover with intricate features [60].

(4) The comprehensive application of multiple remote sensing classification feature variables is an effective way to improve classification accuracy [61]. In this study, a combination of spatial feature parameters of NDRE_705_-PSRI (SFPs), phenological feature parameters (PFPs), and the original time series of NDRE_705_ + PSRI were integrated to create three distinct sets of features. These sets were employed for large-scale agricultural land classification, with findings indicating that PFPs enhanced the accuracy of agricultural land classification more effectively than SFPs. The highest accuracy for agricultural land classification was attained when both feature parameters were incorporated simultaneously, aligning with the findings of a relevant study conducted by Xiao et al. [31]. In this study, agricultural land classification was crop-centric, with the biochemical composition [62] and canopy structure [63] playing pivotal roles in the remote sensing classification of crops. Further exploration and validation are required to assess the impact of these characteristics on the accuracy of remote sensing classification of agricultural land.

(5) Upon comparing the recognition effects of the four types of agricultural land, it was evident that the winter wheat–summer maize rotation area exhibited the highest recognition accuracy, a result consistent with the findings of studies by Dong et al. [53] Zhang et al. [64]. The facility vegetable field ranked second due to its unique time series REVI curve resulting from periodic mulching. Conversely, the identification accuracies for single-season crop fields and orchards were relatively lower, primarily attributed to the similarity in the trends of their REVI time series curves and the limited differentiation in phenological characteristics information. Future studies will incorporate pixel shape indices [65] or employ more refined land class divisions [66] to enhance the accuracy of agricultural land classification.

(6) Remote sensing classification of farmland faces multiple sources of uncertainty that significantly impact classification accuracy. Primary challenges include data loss and spectral distortion from cloud coverage, variations in reflectance characteristics and shadow effects in complex terrain, mixed pixel issues arising from farmland fragmentation, and accuracy assessment complications due to spatially imbalanced validation samples [67,68,69]. Studies have demonstrated that multi-year time series imagery can substantially mitigate uncertainties in crop and farmland classification, particularly through the incorporation of stable temporal indicators like key phenological features and growing season patterns, which enhance the spatiotemporal robustness of classification [70,71,72]. This study effectively reduced classification uncertainty through a comprehensive approach integrating time series red-edge vegetation index features (September 2018–October 2019), robust crop phenological indicators, and a terrain-adaptive parameter optimization system. Nevertheless, the extensive study area posed challenges in obtaining uniformly distributed, spatially representative, and temporally consistent ground validation samples, introducing some uncertainty into the classification results. Future research directions will prioritize the development of a multi-source collaborative validation framework, synthesizing data from mobile field collection, high-resolution remote sensing imagery, and UAV observations to establish a comprehensive, multi-scale validation sample database. Additionally, the implementation of deep learning models will focus on enhancing algorithmic generalization across diverse regions, aiming to simultaneously improve both the accuracy and stability of farmland classification.

## 5. Conclusions

Based on time series Sentinel-2 data (September 2018 to October 2019), this study developed and evaluated an advanced agricultural land classification framework. This framework enables rapid and precise spatial distribution mapping of high-resolution agricultural land types in Shandong Province. The main conclusions are as follows:

(1) We developed an innovative multi-level feature enhancement framework that synergistically integrates NDRE705 and PSRI indices from time series Sentinel-2 imagery. This dual-index approach demonstrated superior performance over traditional single-index methods by capturing complementary aspects of vegetation characteristics throughout the growing season.

(2) The framework’s effectiveness was significantly enhanced through the novel integration of spatial feature parameters (SFPs) and phenological feature parameters (PFPs), which amplified critical temporal characteristics often overlooked in conventional approaches. The optimal classification scheme (FC6), incorporating both enhanced features, achieved 93.21% accuracy (Kappa: 0.9159), representing a 4.83% improvement over baseline methods.

(3) The practical application of this framework successfully mapped 120,996 km^2^ of agricultural land in Shandong Province, accurately differentiating winter wheat–summer maize rotation areas (39.44%), single-season crop fields (36.16%), orchards (14.49%), and facility vegetable fields (9.91%).

This comprehensive approach advances the field by providing a robust, scalable solution for high-resolution agricultural land classification, particularly valuable for regions with complex farming systems and diverse crop patterns. The methodology’s success in capturing subtle spatial patterns and phenological transitions offers new possibilities for improved agricultural land monitoring and management.

## Figures and Tables

**Table 1:** Sentinel-2 band information.

Bands	Resolution (m)	Center Wavelength (nm)	Description Information
B1	60	443.9	Aerosol
B2	10	496.6	Blue
B3	10	560	Green
B4	10	664.5	Red
B5	20	703.9	Red Edge 1
B6	20	740.2	Red Edge 2
B7	20	782.5	Red Edge 3
B8	10	835.1	NIR1
B8A	20	864.8	NIR2
B9	60	945	Water vapor
B11	20	1613.7	SWIR1
B12	20	2202.4	SWIR2
QA60	60	-	-

**Table 2:** Implication of the phenological characteristics parameters.

Phenological Parameters	Implication
Growth rate	Rate of change from valley to peak of NDRE_705_ (NDGR) Rate of change from peak to valley of PSRI (PSGR)
Decay rate	Rate of change from peak to valley of NDRE_705_ (NDDR) Rate of change from valley to peak of PSRI (PSDR)
Growth season amplitude	Differential value between peak value and valley value in growing season (NDGSA, PSGSA)
Growing season cumulative value	The sum of time series spectral indices in the growing season (NDGSC, PSGSC)
Annual cumulative value	The sum of time series spectral indices in the whole phenological period (NDAC, PSAC)

**Table 3:** Construction of classification feature sets.

Feature Sets	Characteristic Variable
FC1	NDRE_705_ Time series
FC2	PSRI Time series
FC3	NDRE_705_ Time series and PSRI Time series
FC4	Optimal Time series Classification Set and Preferred SFPs
FC5	Optimal Time series Classification Set and Preferred PFPs
FC6	Optimal Time series Classification Set and SFPs and Preferred PFPs

**Table 4:** Statistics of phenological feature parameters.

Types of Phenological Characteristic	Parameters
Growth rate	NDGR Oct-Apr
	NDGR Jun-Aug
	NDGR Mar-Aug
	PSGR Oct-Apr
	PSGR Mar-Jul
	PSGR Mar-Sept
	PSGR Jun-Aug
Decay rate	NDDR Apr-Jun
	NDDR Aug-Oct
	NDDR Sept-Nov
	PSDR Aug-Oct
	PSDR Sept-Oct
	PSDR Sept-Mar
Growth season amplitude	NDGSA Oct-Apr
	NDGSA Mar-Aug
	NDGSA Jun-Aug
	NDGSA Aug-Oct
	PSGSA Sept-Oct
	PSGSA Oct-Apr
	PSGSA Sept-Mar
	PSGSA Apr-Jun
	PSGSA Apr-Sept
Growing season cumulative value	NDGSC Oct-May
	NDGSC Mar-Oct
	NDGSC Jun-Oct
	NDGSC Jul-Sept
	PSGSC Nov-May
	PSGSC Apr-Sept
	PSGSC Jun-Oct
	PSGSC Jul-Sept
Annual cumulative value	NDAC Oct-Oct
	PSAC Oct-Oct

**Table 5:** Recognition accuracy of classification feature sets FC4, FC5, and FC6.

	Types	Winter Wheat/Summer Maize	Single Crop Field	Orchard	Facility Vegetable Field	Water	Construction Land	Forest and Grassland	Bare Land	OA%	Kappa
Feature Set											
FC4	UA%	96.77	88.28	89.12	95.89	100.00	81.13	65.52	79.17	89.5	0.8757
	PA%	100.00	85.33	87.33	93.33	96.00	86.00	76.00	76.00		
FC5	UA%.	93.67	88.82	89.40	95.17	97.96	79.25	81.25	86.36	90.38	0.8858
	PA%	98.67	90.00	90.00	92.00	96.00	84.00	78.00	76.00		
FC6	UA%	98.67	94.59	91.45	95.97	97.96	81.48	79.25	91.11	93.13	0.9185
	PA%	98.67	93.33	92.67	95.33	96.00	88.00	84.00	82.00		

## Data Availability

Data are contained within the article.
